# Optoacoustic Sensing of Surfactant Crude Oil in Thermal Relaxation and Nonlinear Regimes

**DOI:** 10.3390/s21186142

**Published:** 2021-09-13

**Authors:** Pavel Subochev, Alexey Kurnikov, Ekaterina Sergeeva, Mikhail Kirillin, Ivan Kapustin, Roman Belyaev, Alexey Ermoshkin, Alexander Molkov

**Affiliations:** Institute of Applied Physics of the Russian Academy of Sciences, 46 Uljanova St., 603950 Nizhny Novgorod, Russia; pavel.subochev@gmail.com (P.S.); sergeeva_ea@ipfran.ru (E.S.); mkirillin@yandex.ru (M.K.); kia@ipfran.ru (I.K.); belyaev@ipfran.ru (R.B.); eav@ipfran.ru (A.E.); molkov@ipfran.ru (A.M.)

**Keywords:** optoacoustic probing, optical absorption, thermal relaxation, Grüneisen parameter, surfactant films, crude oil, laser induced surface deformation

## Abstract

We propose a laser optoacoustic method for the complex characterization of crude oil pollution of the water surface by the thickness of the layer, the speed of sound, the coefficient of optical absorption, and the temperature dependence of the Grüneisen parameter. Using a 532 nm pulsed laser and a 1–100 MHz ultra-wideband ultrasonic antenna, we have demonstrated the capability of accurate (>95%) optoacoustic thickness measurements in the 5 to 500-micron range, covering 88% of slicks observed during 2010 oil spill in the Gulf of Mexico. In the thermal relaxation regime of optoacoustic measurements, the value of optical absorption coefficient (30 mm^−1^) agreed with the data of independent spectrophotometric measurements, while the sound speed (1430 m/s) agreed with the tabular data. When operating in a nonlinear regime, the effect of local deformation of the surface of the oil film induced by heating laser radiation was revealed. The dose-time parameters of laser radiation ensuring the transition from the thermal relaxation regime of optoacoustic generation to nonlinear one were experimentally investigated. The developed OA method has potential for quantitative characterization of not only the volume, but also the degree and even the type of oil pollution of the water surface.

## 1. Introduction

The problem of the constantly increasing pollution of the world ocean requires the development of new approaches for the rapid identification of pollution foci and their rapid analysis. An important indicator of pollution—biogenic (abnormally strong algal blooms) and anthropogenic (industrial waste, domestic waste water, discharges and spills of oil products)—are slicks, overwhelmingly associated with films of surfactants (surfactants) on the water surface. They can be observed with the help of modern ship, air, and space-based facilities, allowing preliminary information on the coverage area and propagation dynamics to be obtained. Basically, for this, methods of active and passive location of optical and radio bands are used [1]. Some of these methods allow the estimation of film thicknesses. Thus, purely optical methods are effective for detecting the thinnest films with a thickness of less than 1 micron by their iridescent appearance; all other observed shades are subjective and can be perceived ambiguously (for example, silver and metallic sheen). The surface brightness spectra in the visible range do not have regular spectral features, but those corresponding to carbon–hydrogen bonds are present in the images of oil films and emulsions in the near-IR range. The corresponding absorption bands are sensitive to the thickness of the film or the layer of oil emulsion, which is the basis for the method of mapping the oil spill in relative units. Their conversion to absolute units is possible with the implementation of accompanying laboratory measurements with samples of oil films taken at sea. The proposed approach makes it possible to obtain estimates of film thicknesses in a wide range—from microns to tens of millimeters, which makes this method one of the most efficient and informative methods for detecting and monitoring large oil leaks using air or space-based facilities. Certain hopes are pinned on radar methods as all-weather instruments for diagnosing the sea surface. However, their effectiveness increases only in the case of using multifrequency radar, while the corresponding devices are still represented by single samples [2]. When using light aircraft and the navy, promising results are achieved with the use of laser technology, as well as through its combination with acoustic systems. One of the new hybrid methods that combines the advantages of optics and acoustics is considered in this work.

The method of pulsed optical-acoustic (OA) diagnostics consists of broadband detection of ultrasonic waves generated as a result of thermoelastic expansion of light-absorbing volumes of the medium when exposed to nanosecond laser radiation [3]. Due to the high optical absorption of hemoglobin in the visible range of optical wavelengths (in comparison with other biological chromophores), the method has found wide application in medicine for OA imaging of blood vessels [4], although some environmental applications of OA technique have also been proposed [5]. While to perform OA diagnostics of single measurement volume it is necessary to illuminate this volume with the laser pulse, the development of laser technologies makes it possible to carry out subsequent OA measurements at laser pulse repetition rates exceeding 1 kHz [6]. At these frequencies, the manifestation of the so-called nonlinear effect becomes possible [7], which consists of a change in the shape and amplitude of the second and subsequent OA pulses associated with the heating of the medium under study due to the absorption of the first laser pulse and incomplete relaxation of the temperature of the medium to the baseline level before exposure to the second [8] and subsequent [9] laser pulses. If the recorded nonlinear distortions of OA pulses are caused by the known temperature dependence of the Grüneisen parameter, the measurements of nonlinear distortions can be used to map the internal temperature of the object under study [10].

In our work, we consider the applicability of optoacoustic methodology [3,8] for detecting crude oil on the water surface, for measuring the thickness of the surfactant layer and for comprehensively characterizing light-absorbing pollutant by its optical and thermophysical properties [1].

## 2. Materials and Methods

### 2.1. Theoretical Model

**Figure 1:**
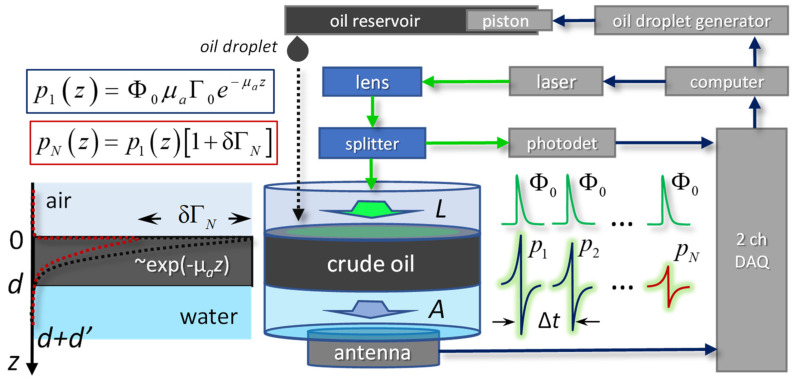
Optoacoustic characterization of oil.

Consider an optically thick layer of crude oil with a thickness $d\gg \frac{1}{\mu_{a}},$ where $\mu_{a}$ is the optical absorption coefficient. Imagine that a layer of oil is evenly distributed over a layer of water with a thickness d′ (Figure 1). From the air to the surface of the oil film z=0, laser probing is carried out in the form of a sequence of N identical pulses with a repetition period Δt. The parameters of each pulse are the duration δt, energy $E_{0}$ and diameter of the light beam L. The response train of N OA pulses is recorded by an ultrasonic sensor located at the bottom of the cuvette with an aperture A and a receiving frequency range $f\in \left[f_{min},f_{max}\right]$.

The pressure increment from the first laser pulse $p_{1}\left(\tau \right)$ recorded by the ultrasonic antenna can be estimated using formula (1), which is a solution to the wave equation with initial conditions in the form of heat sources in the lower half-space z>0:

(1) $$p_{1}\left(\tau \right)=\frac{1}{2}\Phi_{0}\mu_{a}\Gamma \left[\exp\left(c\tau \mu_{a}\right)\theta \left(-\tau \right)-\exp\left(-c\tau \mu_{a}\right)\theta \left(\tau \right)\right],$$

where $\Phi_{0}=4E_{0}/\pi L^{2}$ is the film surface irradiance; $\Gamma =\beta c^{2}/C_{p}$ is the dimensionless Grüneisen parameter at room temperature $T_{0}$, expressed in terms of temperature-dependent parameters; β is the coefficient of thermal expansion; c is the speed of sound; $C_{p}$ is the heat capacity at constant pressure; θ(τ) is the Heaviside function; and τ=t−z/c is the time parameter of the traveling wave.

For the applicability of formula (1), the following conditions must be met for the parameters of the measuring system and the oil film (2)–(7):

(2) $$f_{min}\ll c\mu_{a}\ll f_{max},$$

(3) $$d\gg \frac{1}{\mu_{a}},$$

(4) $$\mu_{a}\gg \mu_{s}\prime ,$$

(5) $$\delta t\ll \frac{1}{\left(4\alpha \mu_{a}{}^{2}\right)},$$

(6) $$\left(d+d\prime \right)\ll A^{2}\mu_{a},$$

(7) $$\frac{2E_{0}\mu_{a}\Gamma }{\pi L^{2}}>NEP,$$

where $\alpha =k/\left(\rho C_{p}\right)$ is the thermal diffusivity of oil, with k being thermal conductivity and ρ being density, $\mu_{s}\prime$ is the coefficient of reduced optical scattering, and NEP is the Noise Equivalent Pressure of the acoustic detector in its [$f_{min},f_{max}$] frequency band. The fulfillment of condition (2) is necessary for the characteristic frequencies generated by the oil slick to belong to the working frequency band of the ultrasonic antenna. Consideration of optically thick (3) and strongly absorbing (4) films makes it possible to significantly simplify Equation (1). For a sufficiently short laser pulse duration (5), thermal relaxation from the region of laser illumination can be neglected. Small distances from the film surface to the antenna (6) allow us to neglect the diffraction effects. The condition characterizing the detectability of oil by the proposed OA method (7) remains valid not only for optically thick (3), but also for thinner oil films whose thickness d exceeds the axial spatial resolution of the acoustical detector.

OA pressure recorded from a laser pulse with a number N can be estimated using formula (8), which is valid when the condition (9) for the duration of a laser pulse train is satisfied and also in the absence of a temperature dependence of the optical absorption coefficient (10):

(8) $$p_{N}\left(\tau \right)=p_{1}\left(\tau \right)\left[1+\delta {\Gamma^{\prime }}_{N}\right],$$

(9) $$N\Delta t<\frac{1}{\left(4\alpha \mu_{a}{}^{2}\right)},$$

(10) $$\mu_{a}\left(N\Delta T\right)=\mu_{a\;N}=const.$$

In case of (9) and (10), the shape of the Nth laser pulse should repeat the shape of the first laser pulse with an accuracy of $\delta {\Gamma^{\prime }}_{N}$, the relative change in the Grüneisen parameter of oil (11) when it is heated from temperature $T_{0}$ to temperature NΔT:

(11) $$\delta {\Gamma^{\prime }}_{N}=\frac{\Gamma \left(T_{0}+N\Delta T\right)-\Gamma \left(T_{0}\right)}{\Gamma \left(T_{0}\right)},$$

where ΔT is the temperature increment on the surface of the oil film (12), arising from the absorption of a single laser pulse:

(12) $$\Delta T=\frac{\Phi_{0}\mu_{a}}{\rho C_{p}}.$$

When performing OA measurements within the considered geometry (Figure 1), it is convenient to calculate the correlation coefficient $R_{N}$, characterizing the applicability of the model (1)–(10).

(13) $$R_{N}={\langle (\frac{p_{1}\left(\tau \right)-\langle p_{1}\left(\tau \right)\rangle }{\langle p_{1}{}^{2}\left(\tau \right)\rangle })(\frac{p_{N}\left(\tau \right)-\langle p_{N}\left(\tau \right)\rangle }{\langle p_{N}{}^{2}\left(\tau \right)\rangle })\rangle |}_{\tau \in \left(-1/\mu_{a}c,0\right)}.$$

The case $R_{N}=1$ corresponds to the fulfillment of conditions (2)–(7), (9)–(10) and allows the possibility of calculating the temperature-dependent parameter $\delta \Gamma_{N}$ according to the simplified formula (8).

In cases where conditions (9) and (10) are not met and $R_{N}<1$, OA measurements become more complicated and require the determination of the temperature-dependent optical absorption coefficient $\mu_{a\;N}$, which in turn requires the determination of the temperature-dependent speed of sound $C_{N}$.

Experimental determination of the temperature dependence of the sound speed $C_{N}$ in crude oil can be carried out by the formula:

(14) $$C_{N}=\frac{d+d\prime }{\tau_{max}^{P_{N}}-\tau_{max}^{PD_{N}}},$$

where $\tau_{max}^{p_{N}}$ and $\tau_{max}^{PD_{N}}$ are the coordinates of the maxima of the optoacoustic and optical signals from the Nth laser pulse, and (d+d′) is the fixed distance between the surface of the oil film and the detector.

Further, from the measured speed of sound $C_{N}$ (14), it is possible to calculate the temperature dependence of the optical absorption coefficient of oil $\mu_{a\;N}$ as a result of differentiation,

(15) $$\mu_{a\;N}^{{\mathrm{diff}}_{+}}={\langle \frac{1}{c_{N}p_{N}\left(\tau \right)}\frac{\partial p_{N}\left(\tau \right)}{\partial \tau }\rangle |}_{\tau \in \left(-3/\left(\mu_{a}c_{N}\right),\;\tau_{max}^{p_{N}}\right)},$$

(16) $$\mu_{a\;N}^{{\mathrm{diff}}_{-}}={\langle \frac{1}{c_{N}p_{N}\left(\tau \right)}\frac{\partial p_{N}\left(\tau \right)}{\partial \tau }\rangle |}_{\tau \in \left(\tau_{min}^{p_{N}},3/\left(\mu_{a}c_{N}\right)\right)},$$

or integration of experimentally recorded OA pulses,

(17) $$\mu_{a\;N}^{int_{-}}=-\frac{1}{c_{N}}{\langle \frac{1+p_{N}\left(c_{N}\tau \right)/min_{\tau_{min}^{p_{N}}}\left[p_{N}\left(c_{N}\tau \right)\right]}{\int_{\tau_{min}^{P_{N}}}^{\tau }P_{N}\left(c_{N}\tau^{\prime }\right)/min_{c_{N}\tau_{min}^{p_{N}}}\left[p_{N}\left(c_{N}\tau^{\prime }\right)\right]d\tau^{\prime }}\rangle }_{\tau \in \left(\tau_{min}^{p_{N}},3/\left(\mu_{a}c_{N}\right)\right)},$$

(18) $$\mu_{a\;N}^{int_{+}}=-\frac{1}{c_{N}}{\langle \frac{1-p_{N}\left(c_{N}\tau \right)/max_{\tau_{max}^{p_{N}}}\left[p_{N}\left(c_{N}\tau \right)\right]}{\int_{\tau }^{\tau_{max}^{P_{N}}}P_{N}\left(c_{N}\tau^{\prime }\right)/max_{c_{N}\tau_{max}^{p_{N}}}\left[p_{N}\left(c_{N}\tau^{\prime }\right)\right]d\tau^{\prime }}\rangle }_{\tau \in \left(-3/\left(\mu_{a}c_{N}\right),\;\tau_{max}^{p_{N}}\right)},$$

where $\tau_{max}^{p_{N}}$ and $\tau_{min}^{p_{N}}$ are the coordinates of the maximum and minimum of the OA signal from the Nth laser pulse.

The following $\mu_{a\;N}$ calculation algorithm is also of practical interest:

(19) $$\mu_{a\;N}^{{\mathrm{fit}}_{+}}:={{\mathrm{fit}}_{e^{\mu_{a}c\tau }}\left\{p_{N}\left(c_{N}\tau \right)/max_{c_{N}\tau_{max}^{p_{N}}}\left[p_{N}\left(c_{N}\tau \right)\right]\right\}|}_{z\in \left(-3/\mu_{a},\;\;c_{N}\tau_{max}^{p_{N}}\right)},$$

(20) $$\mu_{a\;N}^{{\mathrm{fit}}_{-}}:={{\mathrm{fit}}_{e^{-\mu_{a}c\tau }}\left\{p_{N}\left(c_{N}\tau \right)/min_{c_{N}\tau_{min}^{p_{N}}}\left[p_{N}\left(c_{N}\tau \right)\right]\right\}|}_{z\in \left(c_{N}\tau_{min}^{p_{N}},-3/\mu_{a}\right)},$$

where ${\mathrm{fit}}_{e^{\pm \mu_{a}c\tau }}$ is the procedure for exponential interpolation of the increasing or decreasing sections of the experimentally measured OA signals from the same Nth laser pulse.

Finally, having measured by one of the methods (15)–(20)$\;\mu_{a\;N}$, it is permissible to calculate the parameter $\delta \Gamma_{N}$ characterizing the temperature dependence of the Grüneisen parameter:

(21) $$\delta \Gamma_{N}={\frac{max\left[p_{N}\left(\tau \right)\right]/\mu_{a\;N}-max\left[p_{1}\left(\tau \right)\right]/\mu_{a\;1}}{max\left[p_{1}\left(\tau \right)\right]/\mu_{a\;1}}|}_{\tau \epsilon \left(-3/\left(\mu_{a}c\right),0\right)}.$$

### 2.2. Physical Properties of Crude Oil

Thermophysical properties of crude oil, including at different temperatures, were studied in [11,12] and are shown in Table 1. 

**Figure 2:**
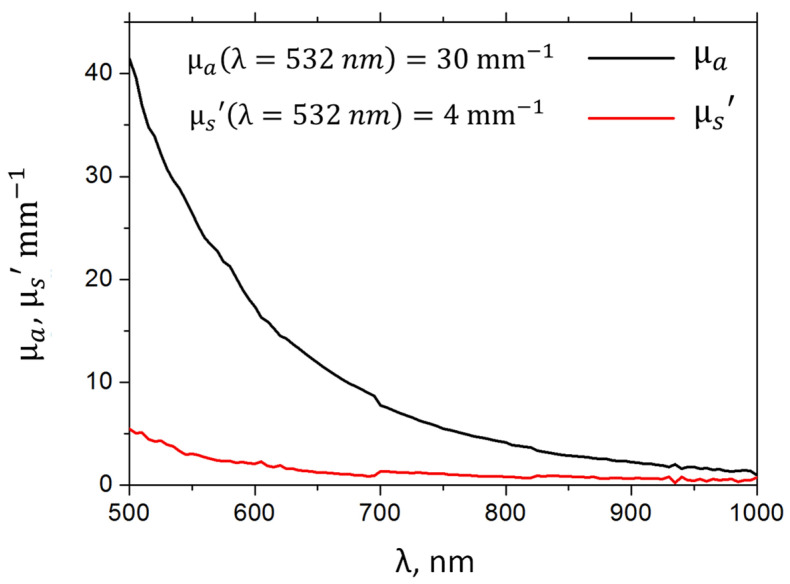
Optical properties of heavy (undiluted) crude oil determined by spectrophotometry measurements.

The determination of optical characteristics was carried out on the basis of spectrophotometric measurements. The sample of heavy crude oil (Lukoil, Russia) was diluted with AI-95 gasoline (Lukoil, Russia) in a ratio of 1:10, after which it was placed in a 1 mm thick quartz cuvette. The collimated transmission, total transmission, and diffuse reflectance of the dilluted sample were measured using a Specord 250 PLUS spectrophotometer (Analytik Jena, Germany) equipped with an integrating sphere. The reconstruction of the spectra of the optical properties of the undiluted sample (reduced scattering coefficient and absorption coefficient) was carried out using an analytical model. The optical properties of crude oil are shown in Figure 2 and confirm the fulfillment of condition (4) in the visible range of optical wavelengths. At 532 nm, the optical absorption coefficient of heavy oil is $\mu_{a}=30\;{\mathrm{mm}}^{-1}$.

### 2.3. Experimental Setup

A diagram of the experimental setup with parameters satisfying the conditions (2)–(7) is shown in Figure 1. To induce OA pulses 532 nm wavelength, the Wedge HB laser (Bright Solutions, Italy) was used with δt=1 ns (5) pulse duration. The generated laser pulse energies $E_{0}$ were controlled using a homemade beam splitter ~1:25 followed by a DET10-A photodetector (Thorlabs, USA). An ultrasonic antenna with an aperture of A=9 mm based on a PVDF-TrFE film with a thickness of 20 μm corresponding to $f_{max}>100$ MHz (2) was used as a receiver of OA pulses. The custom-made matching amplifier provided the lower receiving frequency of the antenna $f_{min}=1$ MHz (2) and NEP of 6 Pa (7). Signals from both acoustic detector and photodetector were digitized by a two-channel 16-bit analog-to-digital converter Razor16 (GaGe, USA) with a sampling rate of 200 MHz.

#### 2.3.1. Oil Film Thickness Measurements

**Figure 3:**
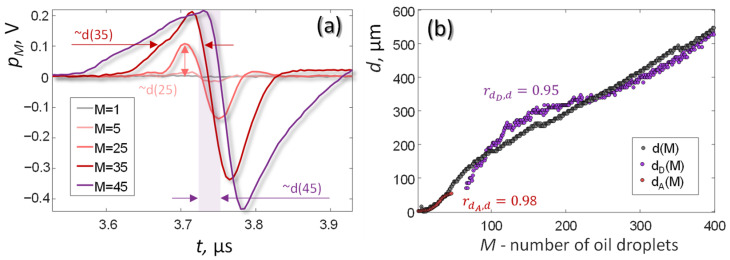
OA measurements of oil thickness. (**a**) OA waveforms for different oil thicknesses. (**b**) The comparison of $d_{A}\left(M\right)$ and $d_{D}\left(M\right)$ with d(M) values (9).

For the OA experiments with variable oil film thickness (Figure 3), the diluted 1:10 *(*$\mu_{a}\sim 3{\;\mathrm{mm}}^{-1}$) sample of crude oil was used. The oil was dropped to the 93 mm diameter cuvette prefilled with $d^{\prime }=5\;\mathrm{mm}$ layer of water. A custom-made oil droplet generator (Figure 1) based on infusion pump Precidor-5003 (Infors, Switzerland) provided M = 400 oil droplets at the maximum rate of 4 droplets per minute. The weight of each droplet, 9 mg, corresponded to the effective increment of the oil thickness, 1.5 µm per droplet. After each controllable change in oil level (OL), the oil film was illuminated by the single optical pulse with $E_{0}=0.4$ mJ energy and L=5 mm laser beam diameter. The OA waveforms recorded by ultrasonic antenna after each oil droplet M were used to evaluate three approaches for OA determination of the oil thickness.

#### 2.3.2. Oil Film Characterization in Thermal Relaxation and Nonlinear Regimes

**Figure 4:**
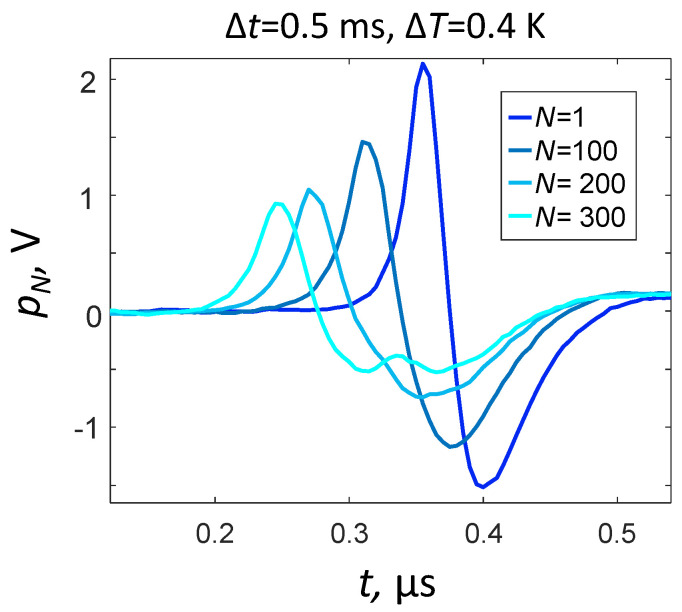
Transition to the nonlinear regime of OA generation at the maximum laser power ΔT=0.4 K and Δt=0.5 ms.

**Figure 5:**
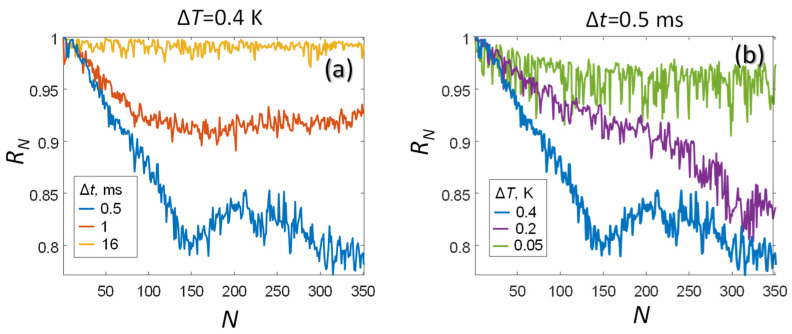
Temperature dependence of $R_{N}$ measured under various conditions of laser exposures characterized by *Δt* and *ΔT*.

**Figure 6:**
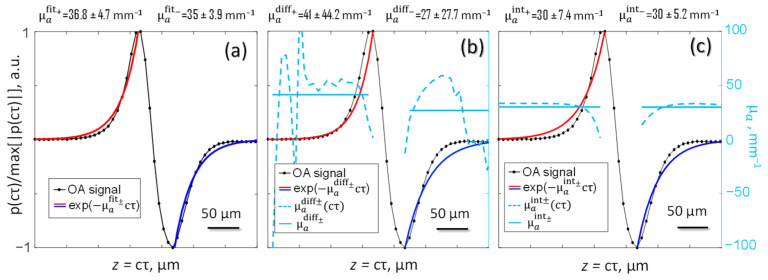
Methods of quantitative OA measurements by means of: (**a**) exponential interpolation, (**b**) differentiation, (**c**) integration of the measured OA signal.

**Figure 7:**
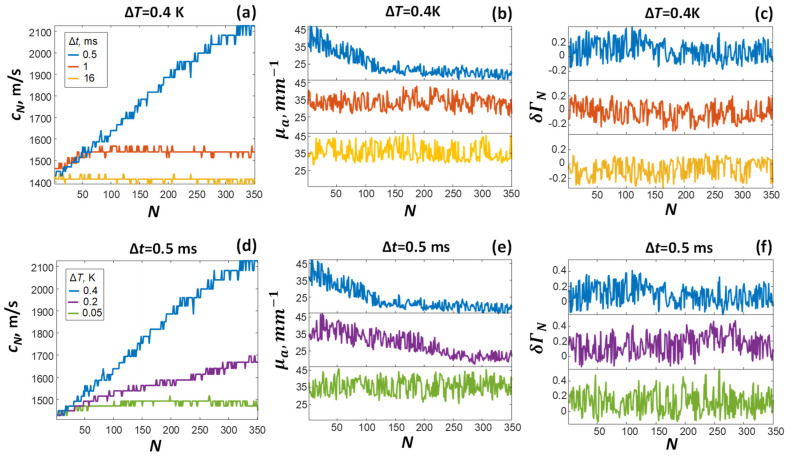
Temperature dependences of $c_{N}$, $\mu_{a\;N}$, $\delta \Gamma_{N}$ measured under various conditions of laser exposures characterized by *Δt* and *ΔT*.

For OA characterization of the optical and thermophysical properties of oil films (Figure 4, Figure 5, Figure 6 and Figure 7), crude heavy oil with $\mu_{a}=30{\;\mathrm{mm}}^{-1}$ was placed directly onto the detector surface ($d^{\prime }=0$), providing an optically thick (3) layer d=0.5 mm. 

To investigate the peculiarities of OA measurements in thermal relaxation and nonlinear regimes, the OA pulses were generated using various preset sequences of the laser pulses N=350. The pulse repetition period was varied in the range from Δt=0.5 ms (nonlinear regime) to Δt=16 ms (thermal relaxation regime). The laser pulse energies were varied from $E_{0}=0.4$ mJ to $E_{0}=0.05$ mJ at a constant laser beam diameter of L=5 mm. Corresponding temperature increments upon absorption of a single laser pulses were, therefore, ranging from ΔT=0.4 K to ΔT=0.05 K. 

Calculations (13)–(21) were carried out for three combinations of Δt and ΔT.

## 3. Results and Discussion

Figure 3a shows typical OA pulse profiles measured from oil films of various thicknesses. For the used parameters of laser radiation, the OA signal from an oil film of minimum thickness (*d* ~1.5 μm or *M* = 1 drop) could not be detected. The minimum detectable oil film thickness was *d* ~4.5 μm (*M* = 3 drops). 

With an increase in the film thickness to *d* ~50 μm (*M* = 35 drops), a directly proportional increase in the amplitude of the OA signals was observed. When the film thickness exceeded the longitudinal resolution of the antenna, a directly proportional increase in the duration of the OA signals was observed. Additionally, a directly proportional shift in the zero of OA pulses was observed, associated with an increase in the oil level in the cuvette.

To recalculate the observed changes in the oil level into changes in the thickness of the oil layer, it is convenient to use formula (14), making the substitutions that are fair in the thermal relaxation mode: $C_{M}\approx C$ and $d\prime \approx c\tau_{max}^{p_{1}}$. Then, we obtain the following formula for calculating the oil thickness, which is valid for any detectable films, including optically thick ones (3):

(22) $$d\left(M\right)=C\left(\tau_{max}^{p_{M}}-\tau_{max}^{PD_{M}}-\tau_{max}^{p_{1}}\right).$$

The results of calculating the oil thickness by formula (22) are shown in Figure 3b by the gray curve. Slight nonlinear distortions of the **d*(*M*)* dependence can be associated with capillary menisci, due to which a small amount of oil can flow from the center of the cuvette and accumulate at the edges. For example, the values of oil thickness *d*(*M* = 400) measured in the center of the cuvette are less than 600 μm (the calculated value without taking into account capillary effects).

The main disadvantage of the method for determining the thickness of the oil film by formula (22) is, however, the need for double measurements. That is, to make accurate measurements of the film thickness *d*(*M*), it is necessary to measure both the delay $\tau_{max}^{p_{M}}$, corresponding to the oil level, and the delay $\tau_{max}^{p_{1}}$, corresponding to the “noise equivalent film thickness” position as a reference value for the water level.

To access OA pulse width, which is necessary for direct measurement of oil thickness, the film should be thinner than the optical penetration and thicker than the spatial resolution of OA sensor. In the corresponding range of oil thickness (~50–500 μm), the pulse-width-based values $d_{D}\left(M\right)\;$ presented in Figure 3b by violet dots agree well (r = 95%) with the results of d(M) measurements (Figure 3b).

The amplitudes of OA pulse can also be used as a source of direct measurement of oil thickness. The values of the oil thickness $d_{A}\left(M\right)$ calculated from the amplitudes of the OA pulses are presented in Figure 3b by red dots and show coincidence with *d(M)* at r = 98% level, although the amplitude-based method of OA thickness measurement also has rather limited range (~5–50 µm) laying between the noise equivalent oil thickness and the axial resolution.

The limits of applicability of all three considered methods of OA thickness measurement are shown in Table 2.

Figure 4 shows the change in the OA signal profile depending on the number of absorbed laser pulses at the maximum laser power ($E_{0}=0.4$ mJ, Δt=0.5 ms). It is easy to see that at N=1, the OA pulse $p_{1}\left(\tau \right)$ has a bipolar shape corresponding to the theoretical model (1). The asymmetry of the experimentally observed OA pulse shape can be associated with the non-ideal reflection of a plane wave at the free air–oil interface, which is not provided by formula (1).

With an increase in the number of absorbed laser pulses (1<N<200), a linear transformation of OA pulses $p_{N}\left(\tau \right)$ is observed, which manifests itself in an increase in pulse duration, increase in effective pulse propagation velocity, and a decrease in the pulse amplitude (Figure 4). Optical absorption of next laser pulses(N>200) leads to nonlinear distortions of the original plane OA wave shape $p_{1}\left(\tau \right)$, especially in the region $p_{1}\left(\tau \right)<0$ more sensitive to diffraction effects [3]. In this case, the characteristic time of transition to the nonlinear transformation of OA pulses is N∆t~100 ms. Figure 5 presents the results of calculating the correlation coefficients $R_{N}$ using formula (13) and allows us to track the processes of linear and nonlinear transformation of the OA pulse shapes $p_{N}\left(\tau \right)$ at various parameters of the laser probing.

When the pulse repetition period Δt=16 (Figure 5a) significantly exceeds the thermal diffusion time (9), the thermal relaxation regime $R_{N}=1$ is realized. In this regime, the heated oil layer manages to transfer heat to the environment before the next laser pulse comes. With a decrease in the pulse repetition period to Δt=1 ms, a thermal equilibrium is established between the processes of laser heating and cooling of oil into the environment during exposure to a series of laser pulses. At the maximum laser power (Δt=0.5 ms, ΔT=0.4 K), after the absorption of N=150 pulses, nonlinear distortions of the $R_{N}$ dependence begin to be observed, which are undesirable from the point of view of the applicability of the considered linear model (1)–(21). It is possible to reduce the influence of nonlinear effects, while maintaining the effect of incomplete thermal relaxation, by reducing the energy of laser pulses to ∆T=0.2 K (Figure 5b). Further weakening of the laser pulse energy to ∆T=0.05 K enhances the thermal relaxation effect, simultaneously decreasing the signal-to-noise ratio for OA signals (Figure 5b), which manifests itself in an increased spread of the corresponding values of $R_{N}$.

To calculate the optical absorption coefficient of oil $\mu_{a\;N}$ at room temperature, it was convenient to use the maximum laser power regime (Δt=0.5 ms, ΔT=0.4 K), achieving the maximum signal-to-noise ratio (Figure 5) by averaging the first N=50 measured OA pulses ($R_{N}>0.95$). The results of substitution of the corresponding averaged values ${\langle p_{N}\left(\tau \right)\rangle }_{N=1..50}$ in formulas (15)–(20) are shown in Figure 6. It is easy to see that the $\mu_{a\;N}$ estimates based on the application of the method (19) and (20) are shifted upward (Figure 6a) relative to the true value (Figure 2). The differentiation procedure (15) and (16) is very sensitive to noise in the original OA signal, which affects the high measurement error (Figure 6b). As for the method of OA signals integration (17)and (18), it showed the best agreement with the results of spectrophotometric measurements (Figure 2 and Figure 6c) and was used in further calculations.

Figure 7 shows the results of OA measurements of $c_{N}$, $\mu_{a\;N}$, and $\delta \Gamma_{N}$ performed in thermal relaxation and nonlinear regimes of laser heating. Application of formula (14) provided a significant increase in the calculated speed of sound ($c_{N}>c_{1}$) in nonlinear regime (Figure 7a,d). After the termination of the laser probing, the calculated values of $c_{N}$ always restored to their original values $c_{1}$. In thermal relaxation regime, the calculated speed of sound did not change ($c_{N}=c_{1}$). It is important to note that the observed directly proportional dependence of the speed of sound on the number of absorbed laser pulses is not typical for oil products. Moreover, according to the results of [13], the increase in oil temperature caused by laser heating had led to the opposite effect—a significant decrease in the speed of sound, $c_{N}$.

Application of formula (17) (Figure 6c) to the measured OA signals provided inversely proportional dependence of the optical absorption coefficient on the number of absorbed laser pulses ($\mu_{a\;N}<\mu_{a\;1}$). The observed effect of “optical clearing” was more pronounced with an increased power of the laser probing corresponding to oil heating. When the laser heating was stopped (Figure 7b,e), the value of the optical absorption coefficient always restored to its initial values ($\mu_{a\;N}=\mu_{a\;1}$). Note that the effect of a “reversible decrease in the calculated optical absorption coefficient of oil during laser exposure” is also not typical for oil products. For example, in [14], with an increase in oil temperature, only a slight decrease in the optical absorption coefficient was observed.

Figure 7c,e shows the results of applying the OA method (21) for $\delta \Gamma_{N}$ calculation. The observed dependences of the Grüneisen parameter on the number of absorbed laser pulses (even under high-power laser irradiation) can also be regarded as an erroneous results, for example, in comparison with the results of [11], demonstrating a significant decrease in the Grüneisen parameter with increasing temperature.

The likely reason for the bias in the estimates of measuring the calculated parameters (14)–(21) is the effect of laser-induced surface deformation occurring at the surfaces of liquids. Even with relatively transparent liquids (water and mineral oil), which allow neglecting the thermal effects, the experimentally observed shapes of laser-curved surfaces seem to be rather difficult to predict theoretically. For example, depending on the radius of the laser beam and the depth of the cuvette, local curvatures of the water surface can take both convex [15,16] and concave [17] shapes. 

According to Figure 4, the characteristic laser-induced displacement of the air–oil interface had a concave shape amounting to at least 150 μm. Taking into account the short propagation path of the OA signal ($d+d^{\prime }=0.5$ mm), the decrease in the thickness of the oil film ($d_{N}<d+d^{\prime }$) could indeed lead to effective increase the calculated (6) speed of sound, leading to the observed discrepancy with literature values (Figure 7a,d). The same laser-induced local curvature at the film surface, which was not considered by the plane-layered model (1)–(10), could also be responsible for the effective decrease in the calculated optical absorption coefficient through an effective increase in the duration of OA pulses recorded by a flat ultrasonic antenna (Figure 1).

For further investigation of the observed effect of laser-induced deformation of the oil film, the free air–oil interface (Figure 1) can be replaced by the fixed interface, such as glass–oil. It is also interesting to experimentally realize the conditions under which the duration of a series of N=350 laser pulses will not exceed the thermal relaxation time of the medium under study. For example, using a more powerful laser with ∆t<0.01 ms instead of ∆t<0.5 ms, it would be possible to measure the temperature dependences of the parameters (14)–(21) using a single train of laser pulses lasting less than one millisecond. By changing the laser irradiance at the sample’s surface, it would probably be possible to change the temperature range N∆T in wide range up to the boiling or even the flash point. 

Consider the prospects for the practical application of the developed OA method. The most obvious one is the complex OA characterization of oil slicks on vast water surfaces, which is important in eliminating the consequences of man-made disasters. According to [18], the overwhelming majority (up to 88% of pieces) of oil slicks during the Deepwater Horizon oil spill was in the range of 5 to 500 microns distinguishable by the OA method. Thus, OA thickness measurement of slicks would be a promising tool for monitoring and predicting the dynamics of large oil spills. At the same time, quantitative OA measurements of the optical absorption coefficient, which linearly depends on the concentration of heavy oil fractions, would seem important from the point of view of controlling the degree of local oil pollution.

Optoacoustic measurements of the temperature dependence of the speed of sound and the Grüneisen parameter are of interest mainly due to the short time required for performing OA measurements in a nonlinear regime. For example, it took only 175 ms to obtain the complete OA data set required to plot the blue curve (Figure 7a). However, the experimentally discovered effect of local laser deformation of an oil film can also have a number of additional practical applications of the developed laboratory OA setup. For example, controlled laser deformations of an oil slick can be used to excite surface waves, which in turn can be used to measure the viscoelastic characteristics of the sample.

A significant practical inconvenience of the developed laboratory OA setup (Figure 1), in comparison with the widely used methods of remote detection and diagnostics of oil pollution [1], is the need for direct contact of the ultrasonic antenna with the sample under study. Thus, to provide OA diagnostics, it is necessary to take a sample from the water surface and place it in the OA cell (Figure 1). The prospect of developing a compact OA device that allows manipulation of the OA cell from the board of a research vessel seems less promising in view of the presence of significant waves on almost any natural water surface. Nevertheless, the prospects for further improvements in the spectral sensitivity of contactless methods for recording OA pulses [19] may actualize the feasibility of developing such portable device.

## 4. Conclusions

We investigated the applicability of laser optoacoustic method for both the detection of oil spills on the water surface and for their comprehensive characterization. The manuscript contains the theoretical basis of the OA method, which allow selecting the technical characteristics of the measuring equipment for the given physical and geometrical characteristics of the crude oil sample. The detailed mathematical apparatus is considered for converting the profiles of the measured OA signals into parameters that are potentially important from a practical point of view of characterizing crude oil pollution: the thickness of the oil layer, the speed of sound, the optical absorption coefficient, and the temperature dependence of the Grüneisen parameter.

The laboratory setup that implements the proposed OA method made it possible to carry out accurate experimental measurements of oil thickness in a wide range, making it possible to characterize the volumes of slicks most often encountered in large man-made disasters. The values of the optical absorption coefficient measured by the developed setup in the thermal relaxation regime of OA pulses generation coincided with the data of independent spectrophotometric measurements, making it possible to quantitatively characterize the degree of surface-active oil pollution. 

By varying the dose-time parameters of laser radiation, the transition from the thermal relaxation regime of optoacoustic generation to the nonlinear one was experimentally investigated. In the nonlinear regime of OA generation, an unexpected effect was discovered, consisting of the local flattening of the oil film under the influence of a heating series of laser pulses.

In general, the results obtained can have good potential for practical application, since the set of characteristics measured by the proposed OA method potentially allows one to quantitatively characterize not only the volume, but also the degree and even the type of oil pollution of the water surface.

## Figures and Tables

**Table 1:** Thermophysical properties of oil.

Physical Property at 20 °C	Variable	Value, Units
sound speed	c	1.4 × 10^3^ m/s
mass density	ρ	865 kg/m^3^
heat capacity	$C_{p}$	1.7 × 10^3^ J/(kg K)
thermal conductivity	k	0.12 W/(m K)
thermal expansion coefficient	β	7.7 × 10^−4^ K^−1^
Grüneisen parameter	$\Gamma =\beta c^{2}/C_{p}$	0.9
thermal diffusivity	$\alpha =k/\rho C_{p}$	8.2 × 10^−8^ m^2^/s

**Table 2:** The limits of applicability of OA methods of oil thickness measurement.

Tested Oil Film Thickness *d*, µm	Duration-Based Indirect OA Measurement of Oil Level d(M)	Amplitude-Based Direct Measurement of Oil Film Thickness $d_{A}\left(M\right)$	Duration-Based Direct OA Measurement of Oil Film Thickness $d_{D}\left(M\right)$
<5 µm	NA	NA	NA
5–50 µm	+	+	−
50–500 µm	+	−	+

## Data Availability

Available upon request.
